# Trends in Human Papillomavirus-Related Health Burden in Greece from 1996 to 2021 with a Focus on Cervical and Lip, Oral Cavity, and Pharyngeal Cancer

**DOI:** 10.3390/pathogens14020197

**Published:** 2025-02-16

**Authors:** Georgios Tampakoudis, Olympia E. Anastasiou

**Affiliations:** 1 Maternal-Fetal Medicine and Obstetrics Saint Luke’s Hospital, 55236 Thessaloniki, Greece; georgetampakoudis@gmail.com; 2 Obstetrics and Gynecology Unit, General Hospital of Goumenissa, 61300 Kilkis, Greece; 3 Institute for Virology, Essen University Hospital, University of Duisburg-Essen, 45147 Essen, Germany

**Keywords:** HPV, cervical cancer, lip, oral cavity, and pharyngeal cancer, oropharyngeal cancer, Greece, papillomavirus

## Abstract

This study aimed to evaluate the burden of HPV-related hospitalization and mortality in Greece, with a focus on invasive cervical cancer and lip, oral cavity, and pharyngeal (LOCP) cancers. A retrospective query using data from the Greek Statistical Office and Eurostat was executed. The query included hospital admission and standardized mortality rates (SDRs) on cervical dysplasia and cervical, vulvar, and vaginal; anal; penile; and LOCP cancers. The hospitalization rate for invasive cervical cancer decreased over time, exhibiting a sharp decrease after 2010, while the hospitalization rate for LOCP cancer decreased after 2011, preceded by a sustained increase from 1996. The hospitalization rate of HPV-attributable diseases in total showed a declining tendency between 2013 and 2017. SDR due to cervical cancer showed a slightly decreasing trend in Greece and the European Union, while SDR due to LOCP cancer showed a slightly increasing trend in Greece, but a decrease in the European Union. The decline in hospitalization rates for HPV-related disease in Greece, especially for cervical cancer and dysplasia, and also the declining SDR for invasive cervical cancer in Greece and the EU, are indications of the positive public health impact of screening programs and the implementation of HPV vaccination.

## 1. Introduction

Human papillomaviruses (HPVs) are a group of viruses capable of causing both benign and malignant lesions on the skin and mucous membranes. Cervical cancer is the most frequent type of cancer caused by HPV, while other HPV-related cancers affect men and women, including anal, female genitourinary tract, mouth/throat, and penile cancers. Over 200 HPV genotypes have been identified, with clinical significance largely determined by their oncogenic potential. HPV-16 is particularly important, as it is found in the majority of HPV-related cancers. Factors that increase the likelihood of HPV-related malignancies include smoking, hormonal contraceptive use, multiple pregnancies, and immunosuppression [1,2,3].

HPV infection remains a major challenge to public health due to its role in cancer development. In 2019, HPV caused an estimated 620,000 cancer cases in women and 70,000 cancer cases in men. Among women, cervical cancer ranked as the fourth most common cancer globally in both incidence and mortality. In 2022, it has been estimated to have produced 660,000 new cases and around 350,000 deaths worldwide, accounting for over 90% of the HPV-related cancers in women [3,4]. Also, in recent years, it has become clearer that HPV plays a crucial role in a substantial number of head and neck cancers, especially oropharyngeal cancers. Each year, head and neck cancers account for over 650,000 cases worldwide, resulting in more than 350,000 deaths, with the incidence increasing in recent years [5]. HPV has also been linked with other malignant and benign conditions, with varying degrees of evidence for the link between HPV infection and the condition. HPV is considered to be a causal agent for benign conditions such as cutaneous and anogenital warts. Also, HPV has been linked to periodontitis, with a recent review concluding that while the results are still conflicting and inconclusive, various studies found an association between oral HPV infection and periodontitis. Focusing on malignant conditions, apart from its known association with cervical cancers; head and neck cancers; and penile, anal, and vulvar/vaginal cancer, HPV infection has been associated with prostate and breast cancer in previous studies. There is some evidence for HPV involvement in the development of prostatitis and the progression from benign to malignant state of prostate tissue. Regarding breast cancer, HPV has been found in malignant tissue; however, HPV as a cause of breast cancer is controversial [6,7].

The progression from persistent infection with high-risk HPV to cervical cancer occurs over an extended period. High-grade cervical dysplasia typically develops 3–6 years after persistent infection, with invasive cancer emerging 10 to over 30 years later. While no reliable data exist on the timeline for cancer development in men, persistent infection is a prerequisite for carcinogenesis. However, not all persistent infections lead to cancer: non-oncogenic HPV types, such as HPV-61, can persist without causing malignancy [2]. Vaccination, safe sex practices, and screening are the pillars of the prevention of HPV infection and HPV-related cancers. The WHO recommends the vaccination of all girls from 9 to 14 years of age before they become sexually active and also the screening of cervical smear for HPV every 5–10 years starting from the age of 30. Also, the vaccination of boys has been recommended and implemented in many countries [3,8].

Data from a 2012 study of 2952 women in Greece showed that HPV could be detected in 50.7% of cervical smears. The most commonly detected HPV types were HPV 51, 53, and 66. HPV detection was linked to factors such as age, time of sexual debut, sexual partner number, and sexual relationship duration while being married or multiparous protected against infection [9]. A more recent study from 2018 involving 2417 women in Greece showed an overall HPV prevalence in cervical smears of 43.9% with high-risk genotypes accounting for 31.3%. The most frequent high-risk type was HPV16 followed by HPV51 and HPV31 [10]. A study published in 2024 that included 253 women demonstrated similar results, with 45.1% of the participants testing positive for HPV. The most common genotype was HPV51, followed by HPV54 and HPV16 [11]. Last but not least, a larger study (GRECOSELF) performed on 12,878 women tested between 2016 and 2018 demonstrated a prevalence of 8.3% for high-risk HPV genotypes in the cervical smear with the detection rate, as the authors noted, “decreasing with age from 20.7% for women aged 25–29 years to 5.1% for women aged 50–60 years”, while “the prevalence of high-grade cervical/vaginal disease or cancer prevalence was 0.6%” [12]. The HPV positivity rates in the above studies are higher than those reported in 2025 meta-analyses containing worldwide data, albeit for women over 50 years of age. HPV prevalence varied geographically, with the highest estimates in Western Africa (32%) and the lowest in Western Europe (6%), while in Southern Europe the prevalence was about 7% [13], which is similar from the rate observed in the GRECOSELF study above.

HPV is the cause of over 80% of oropharyngeal cancers in the United States, but only approximately 3% of oral cavity cancers [14]. There are limited data for the Greek population. Nevertheless, a small study focusing solely on oropharyngeal cancers diagnosed from 2017 to 2022, reported a high HPV-attributable fraction (52%), indicating that HPV as a cause of LOPC cancers is becoming more prevalent. Interestingly, most HPV subtypes detected in positive patients were targeted by the 9-valent HPV vaccine (96.0%), especially HPV-16 (93.3%) [15].

The aim of the present study was to evaluate the burden of HPV-related hospitalization and mortality in Greece with a focus on invasive cervical cancer and lip, oral cavity, and pharyngeal (LOCP) cancer.

## 2. Materials and Methods

### 2.1. Database and Evaluation Criteria

We queried the Greek Statistical Office (ELSTAT) [16] and Eurostat [17] retrospectively to generate the data for our study. The ELSTAT database includes data from all the hospital discharges from 1996 to 2017, including information on the total number of hospital discharges and on the number of discharges grouped by certain primary diagnoses. Primary diagnoses are codified according to the ICD-09-GM in an aggregated manner for the years 1996 to 2012 and data on the total number of hospital discharges, as well as the number of discharges with the primary diagnosis of LOPC cancer and cervical cancer are available. For the years 2013 to 2017, primary diagnoses are codified according to the ICD-10-GM coding system, with data on the total number of hospital discharges and the number of cases, using the following codes as primary diagnosis are available: A63.0 (HPV condylomas), C00 to C14 (lip, oral cavity, and pharynx cancer), C21 (anal cancer), C51 (cancer of the vulva), C52 (vaginal cancer), C53 (cervical cancer), C60 (penile cancer), D06 (carcinoma in situ of the cervix), and N87 (cervical dysplasia). All data are available in an aggregated format.

Based on previous studies, the hospitalization proportion, which can be attributed to HPV from 2013 to 2017, was quantified using the following attributable fractions for each disease group: 26% for LOPC cancers, 50% for penile cancers, 77% for vulvar and vaginal cancers, 88% for cancers of the anus, and 100% for cervical cancer and HPV condylomas [18,19,20]. The total number of HPV-attributable hospitalizations was determined by multiplying the attributable fraction for each condition by the total hospitalizations for that condition and summing the results across all the specified conditions for the years 2013 to 2017.

Data on the Greek population for the years 1996 to 2017 regarding the hospitalization rates were taken from ELSTAT. The hospitalization rate was computed per 100,000 people on the basis of the Greek population size yearly. Data on the standardized death rates (SDRs) for the Greek and European Union (EU) populations were taken from Eurostat for the years 2011 to 2021 for invasive cervical cancer and 1994 to 2021 for LOPC cancer. For the EU population, we used the SDR calculated for the 28 EU countries from 2011 to 2017 for invasive cervical cancer and 2002 to 2017 for LOPC cancer, and the SDR calculated for the 27 EU countries from 2018 to 2021 for both conditions. As noted by Eurostat, “the SDR is the death rate of a population adjusted to a standard age distribution and it is expressed as age-specific mortality rate per 100,000 persons”, with SDR being “calculated as a weighted average of the age-specific death rates of a given population” with “the weights being the age distribution of that population”. Also, SDRs are computed based on the European Standard Population. As the method for standardization, the direct method is applied. SDR has been reported for people between 0 and 64 years (“premature death”), 65 years and more, and for all the population.

### 2.2. Statistics

Statistical analyses were conducted using Excel (Microsoft Office 2019, Redmond, Washington, DC, USA) and the Joinpoint trend analysis software (National Cancer Institute, Bethesda, MD, USA). The time trends were assessed using the Joinpoint model (Joinpoint version 5.02, May 2023) to look into the direction and the intensity of the (linear) trend by computing the annual percent change (APC) and the average annual percent change (AAPC). AAPC is a summary measure of the trend over a prespecified period, describing the average annual percent change (APC) across multiple years. It is calculated as a weighted average of the APCs from the Joinpoint model, with the weights equal to the APC interval length. Linear segments connected at joinpoints, representing the best fit of the observed data, make up the model [21,22]. The threshold of significance was established at *p* < 0.05. The term “stable” was utilized in the event that the APC was between −0.5 and 0.5. In the case of a lower APC, the trends were considered “decreasing”, while in the case of a higher APC, they were considered “increasing”. In all the figures, the asterisk indicates that the APC is significantly different from zero at the alpha = 0.05 level. Ethical review and approval are not required for this study, since it utilized aggregated, publicly available data.

## 3. Results

### 3.1. The Hospitalization Rate for Invasive Cervical Cancer Decreased over Time, Exhibiting a Sharp Decline After 2010

**Figure 1:**
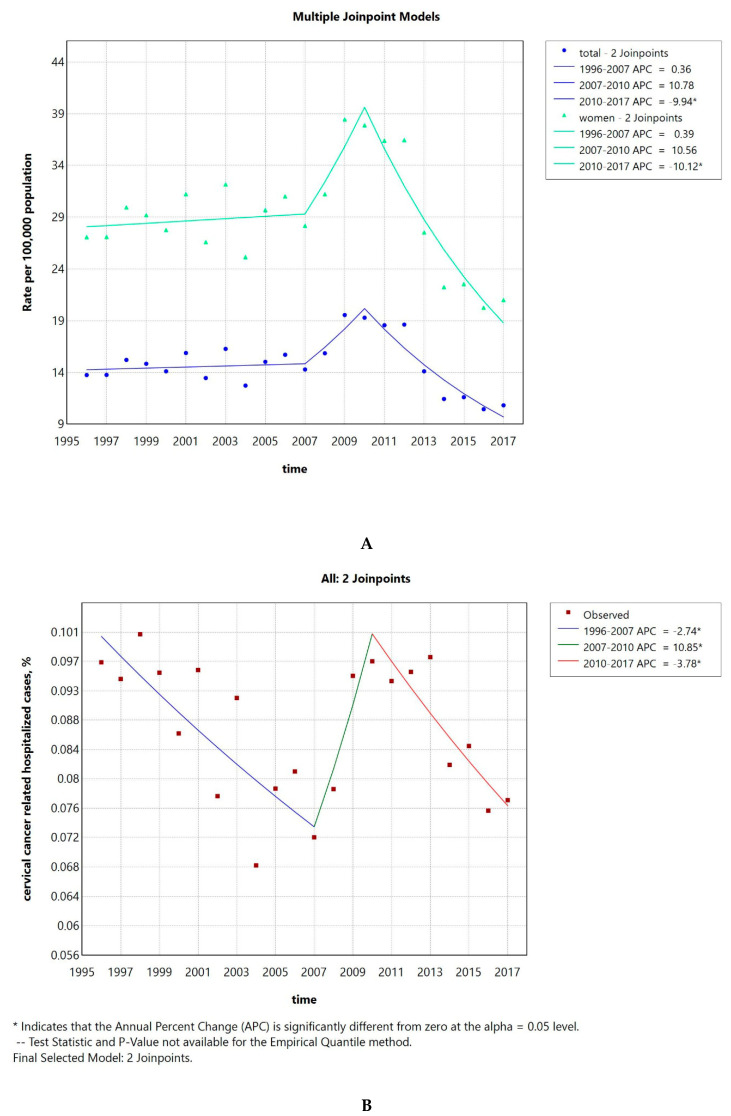
Invasive cervical cancer hospitalization rate per 100,000 inhabitants of both sexes and per 100,000 women from 1996 to 2017 (**A**). Percentage of invasive cervical cancer hospitalized cases to the total number of hospitalizations (**B**).

The hospitalization rate was 14 per 100,000 inhabitants and 27 per 100,000 women in 1996, increasing to 20 and 38, respectively, in 2009 and then dropping to 11 and 21 in 2017. The AAPC was −1.82 (CI −2.9–−0.76) when considering the whole population and −1.9 (CI −2.97–−0.83) when considering only women (Figure 1A). The number of invasive cervical cancer cases compared to the total number of hospitalizations is shown in Figure 1B. The AAPC was −1.26 (CI −1.99–−0.75). Both the AAPC values were significantly different from 0 at the alpha = 0.05 level.

### 3.2. The Hospitalization Rate for LOPC Cancer Decreased After 2011, Preceded by a Previous Sustained Increase

**Figure 2:**
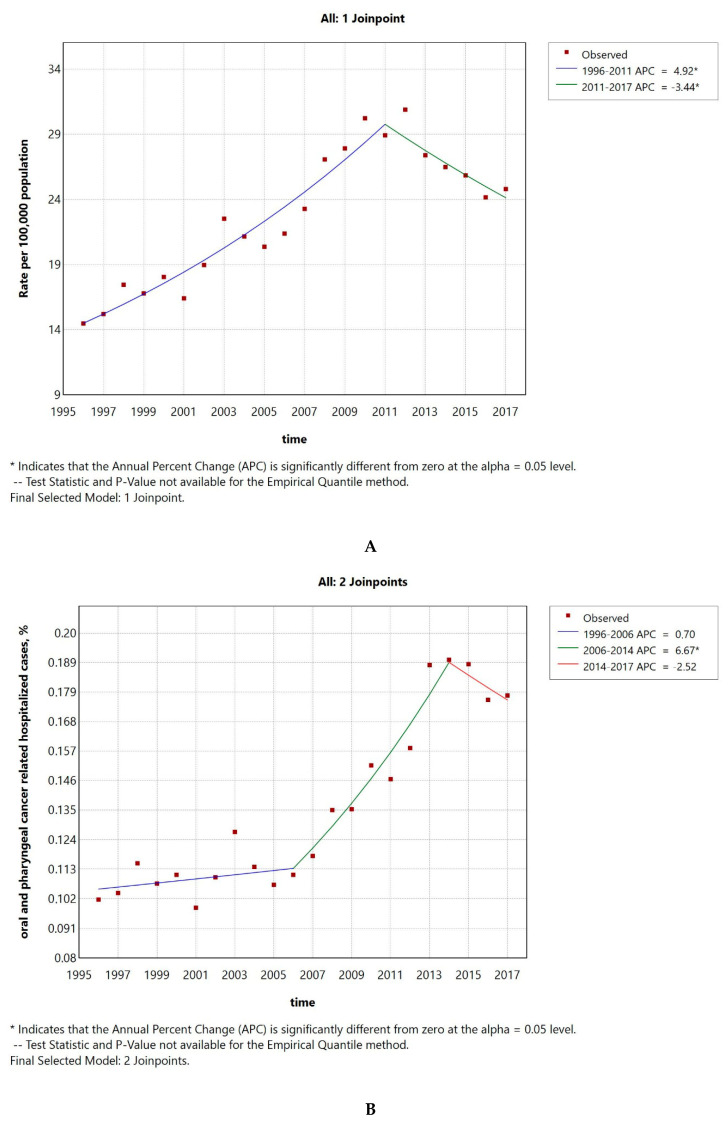
LOPC cancer hospitalization rate per 100,000 inhabitants from 1996 to 2017 (**A**). Percentage of LOPC cancer hospitalized cases to the total number of hospitalizations (**B**).

The hospitalization rate ranged from a minimum of 14 per 100,000 in 1996 to a maximum of 31, while in 2017 it went down to 25. The AAPC was 2.46 (CI 1.86–3) (Figure 2A). The number of LOPC cancer cases compared to the total number of hospitalizations is shown in Figure 2B. The AAPC was 2.46 (CI 1.84–3.06). Both the AAPC values were significantly different from zero at the alpha = 0.05 level.

### 3.3. The Hospitalization Rate of HPV-Attributable Diseases Showed a Declining Tendency Between 2013 and 2017

**Figure 3:**
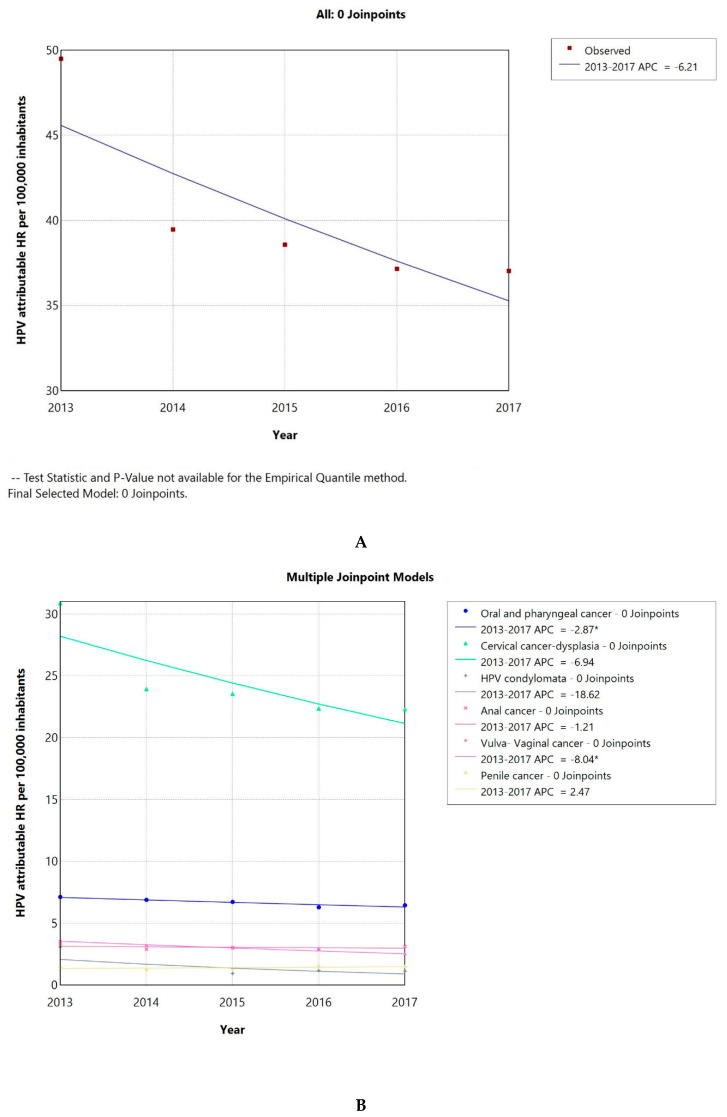

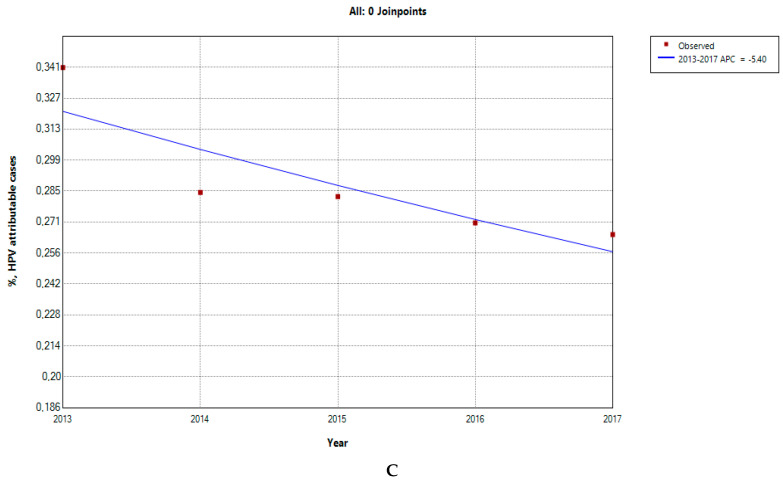
HPV-attributable hospitalization rate per 100,000 people from 2013 to 2017 in total (**A**) and after condition-based stratification (**B**). The percentage of HPV-related hospitalized cases to the total number of hospitalizations (**C**). APC: annual percentage rate. * indicates that the APC is significantly different from zero at the alpha = 0.05 level.

The HPV-attributable hospitalization rate per 100,000 people in Greece has decreased over time, from 50 cases/100,000 inhabitants in 2013 to 37 in 2017. The AAPC was −6.21 (CI −15.92–4.49) (Figure 3A), but it was not significantly different from zero at the alpha = 0.05 level. As seen in Figure 3B, this decline could be primarily linked to a significant reduction in cervical cancer or cervical dysplasia cases. The AAPC for HPV-related conditions is shown in Table 1; all conditions demonstrated a declining trend, except for penile cancer. The AAPC was significantly different from 0, only in the case of LOPC cancer and vulva–vagina cancer. Additionally, the percentage of HPV-related cases to the total number of hospitalizations demonstrated a decreasing tendency with an AAPC of −5.4 (CI −11.05–0.53), which was not significantly different from zero at the alpha = 0.05 level (Figure 3C).

### 3.4. SDR Due to Invasive Cervical Cancer Shows a Slight Decreasing Trend in Greece and the EU

**Figure 4:**
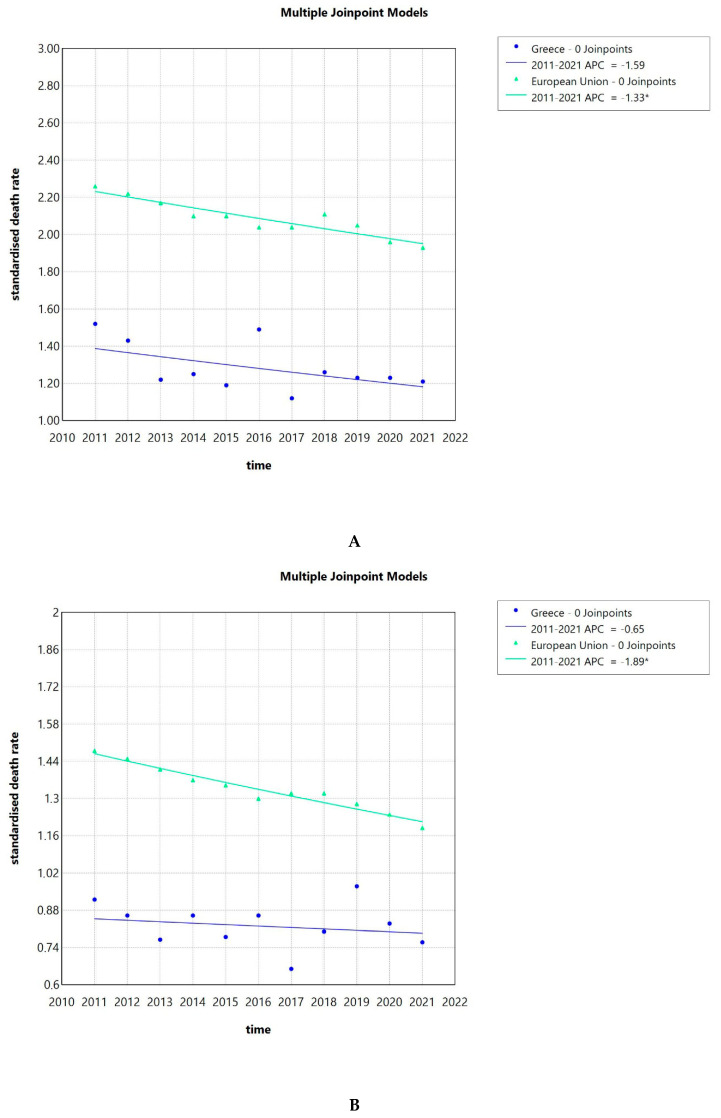

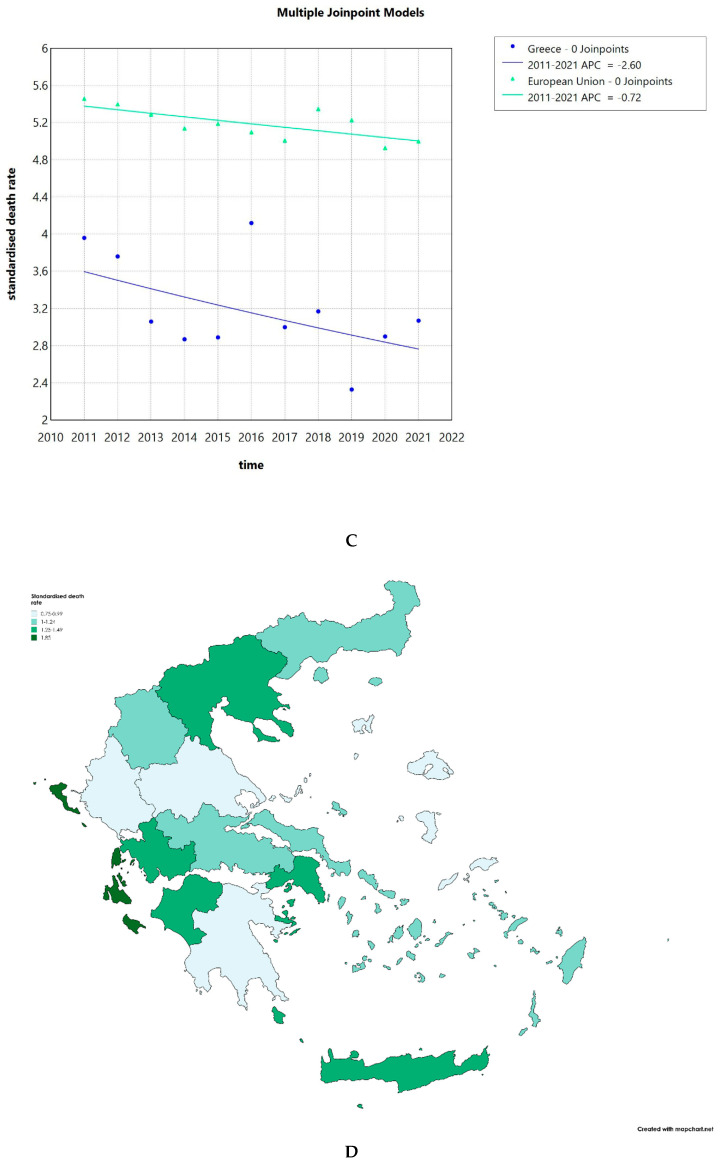
Standardized death rates due to invasive cervical cancer in Greece and the EU from 2011 to 2021 (**A**), standardized death rates due to invasive cervical cancer in Greece and the EU from 2011 to 2021 in the population less than 65 years (**B**), standardized death rates due to invasive cervical cancer in Greece and the EU from 2011 to 2021 in the population 65 years and over (**C**), and standardized death rate, average of the years 2013 to 2021, of the Greek NUTS2 (Nomenclature of territorial units for statistics) (**D**). APC: annual percentage rate. * indicates that the APC is significantly different from zero at the alpha = 0.05 level.

The SDRs due to invasive cervical cancer in Greece were consistently lower than the ones of the EU as a whole. Also, the SDRs in the population 65 years of age and older were about 3 to 4 times higher in Greece and the EU compared to those observed in younger individuals. This effect could be observed for the whole population, as well as the population over and under 65 years of age. From 2011 to 2021, a slightly decreasing tendency could be observed for both the Greek cervical cancer SDR and the overall EU ones as seen in Figure 4A. The AAPC for Greece was −1.59 (−4.32–1.25) and for the whole EU −1.33 (−1.95–−0.72), the latter being significantly different from zero at the alpha = 0.05 level. 

Focusing on the Greek population under 65 years of age, we saw a stagnation of the cervical cancer SDR over time with an AAPC of −0.65 (−3–1.79), while the AAPC for the whole EU was −1.89 (−2.29–−1.48), showing a decreasing tendency. The latter AAPC was significantly different from zero at the alpha = 0.05 level (Figure 4B). When looking into the population of 65 years and over, we observed a declining trend of the cervical cancer SDR for the Greek population with an AAPC of −2.6 (−5.84–0.81), while the AAPC for the whole of the EU was -0.72 (−1.45–0.01) (Figure 4C).

Next, we calculated the average of the cervical cancer SDR for the total population, calculated for the years from 2013 to 2021 for the Greek regions corresponding to NUTS2 (Nomenclature of territorial units for statistics). The average for Greece in total was 1.24 per 100,000 inhabitants. As seen in Figure 4D, the Ionian Islands was the region with the highest average SDR of 1.85, while the lowest was observed for the Peloponnese with 0.84. The Peloponnese, Epirus, Thessaly, North and South Aegean, Sterea Ellada, East Macedonia and Thrace, and West Macedonia had lower rates than the Greek average, while West Greece, Crete, Central Macedonia, Attica, and the Ionian Islands had higher rates than the Greek average.

### 3.5. SDR Due to LOPC Cancer Shows a Slightly Increasing Trend in Greece, but a Decreasing One in the EU

**Figure 5:**
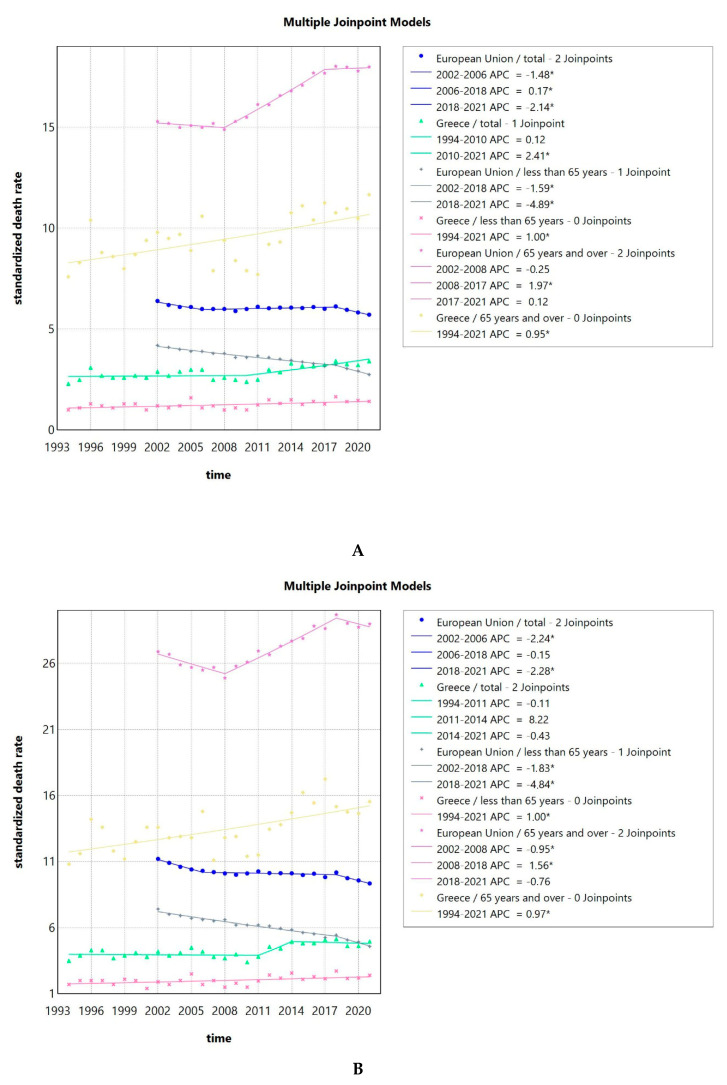

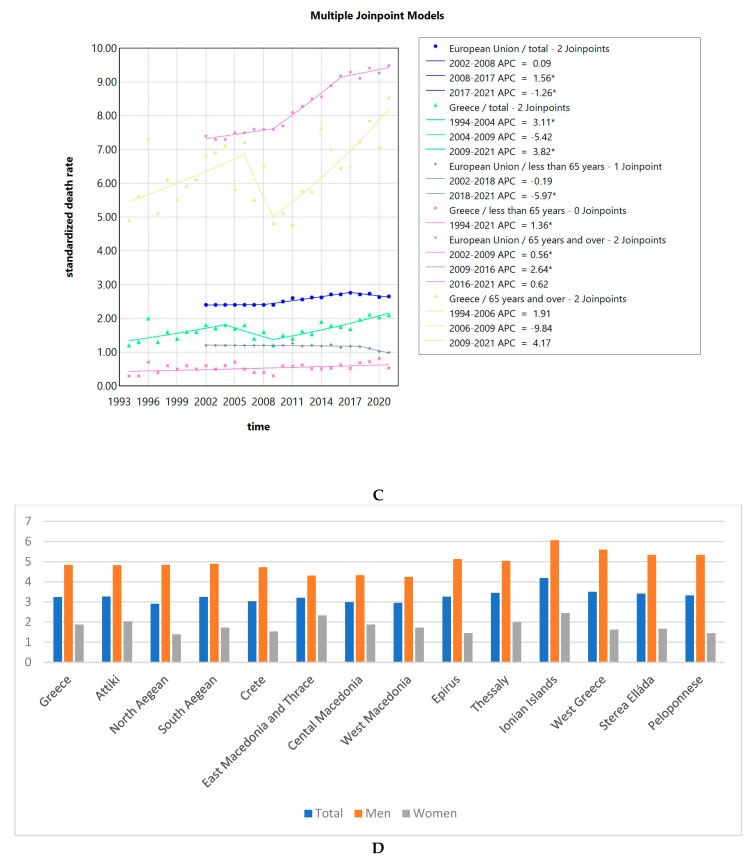
Standardized death rates of both sexes due to LOPC cancer in Greece from 1994 to 2021 and the EU from 2002 to 2021, stratified by age (**A**); standardized death rates of men due to LOPC cancer in Greece from 1994 to 2021 and the EU from 2002 to 2021, stratified by age (**B**); standardized death rates of women due to LOPC cancer in Greece from 1994 to 2021 and the EU from 2002 to 2021, stratified by age (**C**); and standardized death rate, an average of the years 2013 to 2021 of the Greek NUTS2 (Nomenclature of territorial units for statistics) for the total population, men and women (**D**). APC: annual percentage rate. * indicates that the APC is significantly different from zero at the alpha = 0.05 level.

The SDRs due to LOPC cancer in Greece were consistently lower than the EU SDRs as a whole. This effect could be seen observed for the whole population, but also when focusing on women vs. men, with and without stratification for age (less or over 65 years of age) as seen in Figure 5A–C. Looking at both sexes together or men only, older adults (65 years old and over) had a 6 to 10 times higher SDR compared to younger people for the Greek population, while for the population of the EU in total, the SDR was 4 to 6 times higher, respectively. For women, older women (65 years old and over) had an 8 to 19 times higher SDR compared to younger women for the Greek population, while for the EU population, the SDR was 6 to 10 times higher, respectively. Men had higher SDR compared to women at all times. The difference was less pronounced for the Greek population, with men (all ages and over 65 years of age) having a 2–3 times increased risk, while the risk for men less than 65 years compared to women was 3 to 7 times increased. For the EU, the risk for all men was 4–5 times increased compared to all women, 4 times increased for men vs. women over 65 years, and 5–7 times increased for the less than 65 years population of men vs. women.

The trends in SDR for LOPC cancer are shown in Figure 5A. The AAPC for Greece was 1.05 (0.37–1.68) for the total population, 1 (0.35–1.65) for people less than 65 years old, and 0.95 (0.39–1.5) for people 65 years of age and older. The AAPC for the whole EU was −0.55 (−0.66–−0.45) for the total population, −2.12 (−2.34–−1.95) for people less than 65 years old, and 0.88 (0.72–0.99) for people 65 years of age and older. All the values were significantly different from zero at the alpha = 0.05 level.

Focusing on men, the SDR followed a pattern similar to the one for the total population, as seen in Figure 5B. The AAPC for Greece was 0.7 (−0.08–1.6) for all men, 1 (0.22–1.77) for men less than 65 years old, and 0.97 (0.5–1.45) for men 65 years of age and older. For the two latter groups, the AAPC was significantly different from zero at the alpha = 0.05 level. The AAPC for the whole EU was −0.93 (−1.06–−0.82) for all men, −2.31 (−2.69–−2.02) for men less than 65 years old, and 0.39 (0.17–0.55) for men 65 years of age and older, all of which were significantly different from zero at the alpha = 0.05 level.

The trends in SDR for women are seen in Figure 5C. The AAPC for Greece was 1.78 (0.86–2.97) for the total female population, 1.36 (0.06–2.65) for women less than 65 years old, and 1.52 (0.66–2.66) for women 65 years of age and older. The AAPC for the whole EU was 0.49 (0.32–0.63) for the total female population, −1.12 (−1.31–−0.89) for women less than 65 years old, and 1.34 (1.21–1.45) for women 65 years of age and older. All the values were significantly different from zero at the alpha = 0.05 level.

Next, we calculated the average for the LOPC cancer SDR for the total population, for men and women, for the years from 2013 to 2021 for the Greek regions, corresponding to NUTS2 (Nomenclature of territorial units for statistics). The average for Greece in total was 3.24. Men had consistently higher average SDR compared to women. As seen in Figure 5D, the Ionian Islands was the region with the highest average SDR of 4.19. This was also true when considering men (6.07) and women (2.44) separately. The lowest SDR for the whole population was observed in North Aegean with 2.95. North Aegean, Crete, East Macedonia and Thrace, and Central and West Macedonia had lower rates than the Greek average, while Attica, South Aegean, Epirus, Thessaly, the Ionian Islands, West Greece, and the Peloponnese had higher than the Greek average.

## 4. Discussion

The hospitalization rate for invasive cervical cancer decreased over time, exhibiting a sharp decline after 2010. The hospitalization rate was 14 per 100,000 inhabitants and 27 per 100,000 women in 1996, increasing to 20 and 38, respectively, in 2009 and then dropping to 11 and 21 in 2017. The SDRs due to invasive cervical cancer in Greece were consistently lower than the ones of the EU as a whole in our study, which is consistent with the results published before [23]. The SDR due to cervical cancer showed a decreasing trend, both in Greece and the EU, with the AAPC for Greece being −1.59 (−4.32–1.25) and for the whole EU −1.33 (−1.95–−0.72). This trend seems to be a global phenomenon. In a recent analysis including global data, 26 of the 32 analyzed countries had stable or even decreasing temporal trends for incidence and mortality due to cervical cancer, with the authors noting that the effect was more prominent in countries with effective cervical cancer screening programs [24]. Focusing on regions of Greece, we observed that the average SDR due to invasive cervical cancer for the whole country was 1.24 per 100,000 population. The Ionian Islands was the region with the highest average SDR of 1.85, while the lowest was observed in the Peloponnese with 0.84, indicating some interregional variability.

The hospitalization rate in Greece for LOPC cancer decreased after 2011, following a sustained increase over time. Overall, it ranged from 14 per 100,000 population in 1996, to a maximum of 31 per 100,000 population. The AAPC from 1996 to 2017 was 2.46 (CI 1.86–3), demonstrating an overall increasing tendency. The results are largely consistent with those presented in the literature for other countries. A previously published study presenting global data from 1983 to 2002 reported a significant increase in oropharyngeal cancer incidence, predominantly in developed countries and at younger ages [25]. Another recently published study using data from 1993 to 2012 and focusing on the incidence of oropharyngeal and oral cavity cancers worldwide also reported increasing oropharyngeal cancer incidence among many countries. In men, elevated rates of oropharyngeal cancer were increasingly observed in older age groups, which the authors attributed to human papillomavirus-related birth cohort effects. In women, they described more diverse patterns, suggesting a complex interplay of risk factors [26].

The SDR due to LOPC cancer showed a slight increasing trend in Greece, but a decreasing one in the EU. The AAPC for Greece was 1.05 (0.37–1.68) for the total population, while for the whole EU, it was −0.55 (−0.66–−0.45). The SDRs due to LOPC cancer in Greece were consistently lower compared to the ones of the EU as a whole. This effect could be observed for the whole population, but also after stratification according to sex and age. Our results are consistent with those of a previously published study reporting that the mortality rates were decreasing for LOPC cancer in the EU from 1990 to 2019 [27]. Interestingly, another study looking at LOPC cancer incidence and mortality at a global level from 1990 to 2016 reported that “male mortality decreased over the last decades in several European countries, with earlier and sharper declines in southern Europe; conversely, mortality was still increasing in a few eastern European countries and the United Kingdom”. On the contrary, female mortality mildly increased in several countries of Europe. The authors hypothesized that “changes in tobacco and alcohol exposure in men over the last decades were a likely explanation for the favorable trends in LOPC cancer mortality and incidence observed in selected countries worldwide, while increased HPV infection was likely responsible for the rise in oropharyngeal cancer incidence, where observed” [28]. A recent systematic review reported that from 1990 to 2019, low-middle and low socio-demographic regions consistently showed the highest SDR due to LOPC cancer, while regions with high socio-demographic factors exhibited decreasing SDR lip and oral cavity cancer and increasing for other pharyngeal cancers [29]. Focusing on different regions of Greece, the average SDR from 2013 to 2021 for Greece in total was 3.24 per 100,000 population. The Ionian Islands was the region with the highest average SDR of 4.19. This was also true when considering men (6.07) and women (2.44) separately. The lowest SDR for the whole population was observed in North Aegean with 2.95, which indicates here also a small interregional variability. Here, as well as in the case of invasive cervical cancer SDR, the interregional variability amongst different Greek regions is small. Data on the reasons behind these regional differences are scarce. Interestingly, a recent study on the total mortality in different regions of Greece described a “gradual convergence of the mortality rates across the country” as an indicator of a more effective welfare system [30].

The HPV-associated hospitalization rate per 100,000 people in Greece demonstrated a decreasing tendency over time, from 50 cases/100,000 inhabitants in 2013 to 37 in 2017. The AAPC was −6.21 (CI −15.92–4.49), but it was not significantly different from zero at the alpha = 0.05 level. This decline could be primarily linked to a large decline in cervical cancer or dysplasia cases. The AAPC was significantly different from zero, only in the case of LOPC cancer and vulva–vagina cancer for the short time window of 2013 to 2017. Although the time window for which we could calculate the overall hospitalization rate of HPV-attributable diseases in Greece was short, the observed declining trend is consistent with previous studies from other European countries [31,32,33,34]. Focusing on vulva and vaginal cancer data, we observed a steep declining trend for the short time window. The trends for the hospitalization rates for these conditions are less consistent in the literature, with studies reporting an increase, a decrease, or a stable status [31,35,36].

Our study provides valuable information on the burden of HPV-related hospitalization and mortality in Greece. However, the data presented come with certain limitations. Health insurance claims form the basis of our analysis, so our dataset includes only individuals who sought medical advice and were diagnosed with the relevant conditions. The analysis focuses on hospitalization, so no data are included (or are available) per definition about ambulatory care cases, thus it is only an indirect rough measure of the incidence or prevalence of the disease, leading to an underestimation of the overall HPV-related burden. This is especially true for less severe conditions, such as cervix dysplasia or HPV condylomas. Also, our analysis does not include some other conditions that have been associated with a lesser degree and with a lower degree of certainty to HPV, such as laryngeal, esophageal, and skin cancer [1], probably leading to a further underestimation of the overall HPV burden. Furthermore, the HPV-attributable rate for different conditions is computed according to bibliographic estimates and is not the product of a direct examination of biological materials. Another issue is the fact that the overall HPV burden analysis, the cervical or LOPC cancer hospitalization rates, and standardized mortality rates analysis cover overlapping but variable time windows due to a lack of data availability. We aimed to maximize data utilization, which comes at the expense of uniformity and ease of interpretation. Especially, the analysis of the estimated overall HPV burden in Greece covers a limited time span. Lastly, coding errors in the health claim data are a possibility; however, they are expected to be negligible, considering how prevalent these conditions are. Our study has some important strengths. It is, to the best of our knowledge, the first study to present data on HPV-related hospitalization and mortality for the whole Greek population over time. The inclusion of the total Greek population helps us avoid a sample selection bias. The longitudinal character of the study provides evidence of the changes in HPV-related burden over time and allows the formation of hypotheses on the success of the implemented screening and vaccination strategies.

The study has important implications for public health policy in Greece. It also holds value for healthcare providers, especially those involved in preventive medicine. The decline in hospitalization rates for HPV-related disease in Greece, especially for cervical cancer and dysplasia, and also the declining SDR for invasive cervical cancer in Greece and the EU, are indications of the positive public health impact of screening programs and the implementation of HPV vaccination. Greece introduced HPV vaccination to the national vaccination program first in 2008. In the beginning, the vaccine recommendation included only girls, while later the recommendation was expanded to also include adolescent boys. The current recommendation includes the vaccination of adolescent girls and boys from 9 years old, and the HPV DNA screening test of women of 30 years of age or older is recommended every 5 to 10 years, with both strategies aiming at the reduction in cervical cancer incidence and mortality in the general population [37]. Although there are no official data on HPV vaccination coverage in Greece, the coverage is estimated to be around 55%, which is low compared to other countries in the developed world according to a 2024 study [38]. Another study published in 2024 with 2685 participants indicated that while the awareness of the existence of HPV infection is high in the Greek population, the perception of the importance of HPV testing and vaccination is low, indicating that more efforts may be necessary to educate the public [39]. As the vaccination of the male population has started very recently and focuses on adolescent boys and considering the multifactorial nature of LOPC, penile, or anal cancer causes, it is very unlikely that HPV vaccination had any measurable impact on their incidence. Interestingly, HPV vaccine acceptability has been reported to be high despite the lack of vaccine promotion to Greek high-risk men [40]. Also, there is an ongoing campaign to reduce cervical cancer. It includes an examination program, free of charge, which is aimed at all women from 21 to 65 years old who have not been diagnosed with cervical cancer. Importantly, it covers both insured and uninsured women. Younger women, from 21 to 29 years, are entitled to a gynecological examination and cervical smear test every 3 years. Women from 30 to 65 years, who have not had an HPV-DNA test during the previous 5 years and a cervical smear test during the previous 3 years, are entitled to a gynecological examination and HPV-DNA test. For both groups, if specific findings arise from the cytological examination, the patients are entitled to a second visit with colposcopy and biopsy [41].

In summary, our study presents a thorough analysis of trends in HPV-related hospitalization and mortality, with a focus on invasive cervical cancer and LOPC cancer, in the Greek population. The findings highlight the evolving patterns of HPV-related diseases in Greece and provide valuable insights into the potential impact of preventive measures like screening and vaccination. This research enhances understanding of the burden of HPV-related diseases and serves as a useful resource for guiding future public health initiatives in Greece.

## Figures and Tables

**Table 1:** Average annual percent change (AAPC) for HPV-related conditions from 2013 to 2017.

Condition	AAPC	CI
LOPC cancer	**−2.87**	−4.3–−1.42
Cervical cancer/dysplasia	−6.94	−17.9–5.31
HPV condylomas	−18.62	−54.6–45.27
Anal cancer	−1.21	−9.34–7.62
Vulva–vaginal cancer	**−8.04**	−10.46–−5.61
Penile cancer	2.47	−8.66–14.76

Bold indicates an AAPC significantly different from zero at the alpha = 0.05 level.

## Data Availability

Data publicly available in aggregated form, see Materials and Methods section.
